# Ontario Veterinary College

**Published:** 1901-01

**Authors:** 


### COMMENCEMENT EXERCISES.

### ONTARIO VETERINARY COLLEGE.

The Christmas examinations were concluded on Friday, December 21st. The great rise in the price of horses owing to the developments of the South African war and the greater mobility of troops that recent military operations prove to be imperative are conclusive evidence that the demand for horses for the wars of the present day as well as for civilian use will continue to increase. This as well as the great stimulus that has been going on for years in the breeding of pure-bred cattle of all the various types, and also the need for scientific knowledge in relation to the inspection of all our meat- and milk-producing animals, cannot fail to have a marked effect on the veterinary profession and continue to induce young men of good education and ability to follow it. The Board of Examiners, which is composed of prominent veterinary practitioners, awarded diplomas to the following gentlemen :

Robert K. Bryant, Sunderland ; Herbert Killips, Tonawanda, N. Y. Z.; T. McNees, Butler, Pa.; Orange Judd Phillips, Warrensburg, Mo.; W. J. R. Ramage, Mooresburg ; Ira B. Rivenburgh, Chatham, N. Y.; Herbert L. Switzer, Springfield, Mass. Primary examinations in anatomy—Norman A. Anderson, passed primary in anatomy; Thomas H. Monahan, passed primary in anatomy. The college will reopen on January 2, 1901.

